# The Serum Hepatitis B Virus Large Surface Protein as High-Risk Recurrence Biomarker for Hepatoma after Curative Surgery

**DOI:** 10.3390/ijms23105376

**Published:** 2022-05-11

**Authors:** Hung-Wen Tsai, Yun-Ping Lee, Chia-Jui Yen, Kuang-Hsiung Cheng, Chien-Jung Huang, Wenya Huang

**Affiliations:** 1 Department of Pathology, National Cheng Kung University Hospital, College of Medicine, National Cheng Kung University, Tainan 70101, Taiwan; hungwen@mail.ncku.edu.tw (H.-W.T.); ckh774004@gmail.com (K.-H.C.); 2 Department of Medical Laboratory Science and Biotechnology, College of Medicine, National Cheng Kung University, Tainan 70101, Taiwan; fishskin816@gmail.com; 3 Division of Hematology and Oncology, Department of Internal Medicine, National Cheng Kung University Hospital, College of Medicine, National Cheng Kung University, Tainan 70101, Taiwan; yencj@mail.ncku.edu.tw; 4 Department of Internal Medicine, Taipei City Hospital, Taipei 10341, Taiwan; dai62@tpech.gov.tw; 5 Institute of Basic Medical Science, College of Medicine, National Cheng Kung University, Tainan 70101, Taiwan

**Keywords:** chronic hepatitis B, HBV surface antigen, ELISA, hepatocellular carcinoma, prognosis

## Abstract

Chronic hepatitis B (CHB) virus infection is the most important cause of HCC and is also associated with tumor progression. The development of viral biomarkers for HCC prognosis is critical in evaluating relative risks to recurrence in the CHB HCC patients. We report that the large HBV surface protein (LHBS) expression increased in the tumors, implicating that it played a significant role in tumor development. To detect the LHBS in serum and evaluate its association with HCC progression, we developed a sandwich ELISA method for LHBS. The mouse monoclonal antibodies for the pre-S1, pre-S2, and HBS regions were in-house generated and constructed into a chemiluminescent sandwich ELISA system, which allowed sensitive and quantitative measurement of the protein. Using this ELISA assay, we estimated the expression of LHBS in CHB and HCC patients. We found that the serum LHBS level was correlated with the HBS but not the viral titer in serum, indicating that HBV surface proteins’ expression does not mainly depend on viral replication. Moreover, both serum LHBS and HBS levels were lower in the HCC patients than in the CHB. The liver LHBS signals, detected by immunohistochemical staining, showed significant correlations with the serum LHBS and HBS levels. In addition, the more elevated serum LHBS but not HBS level was significantly associated with cirrhosis and worse disease-free and overall survival rates, based on the multivariate analysis. Conclusion: LHBS plays a specific role in tumor progression and is an independent parameter associated with HCC recurrence. Serum LHBS represents a novel noninvasive biomarker for HCC patients with a worse prognosis after surgery.

## 1. Introduction

Chronic hepatitis B (CHB) virus infection is the most important cause of hepatocellular carcinoma (HCC) in many regions of the world. Currently, 2 billion people have been infected with HBV, and 350 million of them have CHB. HBV infection also results in 500,000 to 1.2 million deaths per year caused by chronic hepatitis, cirrhosis, and HCC, and is among the top 10 leading causes of death worldwide [1,2,3]. Because the responses to adjuvant chemotherapies and radiation are usually not good in HCC, tumor recurrence rates after curative hepatic resection are high [4]. Therefore, long-term monitoring of HCC progression after primary treatments is important. The identification of effective and reliable high-risk biomarkers for HCC progression remains a major challenge.

HBV oncoproteins have long been known to play a critical role for HCC progression [3]. Viral factors not only trigger pathological changes that lead to carcinogenesis but also enhance the proliferation and recurrence of tumors after primary treatments. Antiviral therapies have been shown to benefit HCC prognosis after primary cancer treatments such as surgery, radiofrequency ablation (RFA), and transarterial chemoembolization (TACE), suggesting that viral factors are consistent oncogenic players in multiple stages of HCC development [5,6]. Therefore, it is promising to identify high-risk HBV-related markers for recurrence that can be monitored frequently after primary treatments. The oncogenic HBX protein has long been shown to be closely related to HCC development by directly interacting with basal transcription factors and the acetyltransferase CBP/p300 [7]. HBX functions as a transcriptional transactivator of different host genes involved in cellular proliferation control, chromatin remodeling, and cell cycle progression [8,9]. Some studies have reported that HBX gene mutations are correlated with the prognosis of HCC, probably through their aberrant interactions with host carcinogenic pathways [10,11]. However, despite its significant role in HCC progression, HBX is rarely detectable in peripheral blood, compromising the feasibility of its use as a convenient biomarker for monitoring HCC patients after primary treatments.

Recent studies have also found that the large HBV surface (LHBS) protein is highly associated with HCC development [12]. LHBS contains the integral pre-S1, pre-S2, and S domains and binds to the HBV receptor sodium taurocholate co-transporting polypeptide (NTCP) on hepatocytes to facilitate viral entry and morphogenesis [13,14]. The accumulation of viral LHBS and its prevalent pre-S mutants in chronic HBV carriers triggers a sustained endoplasmic reticulum (ER) overload response, leading to ER stress-mediated cell proliferation, metabolic switching, and genomic instability, which are associated with pro-oncogenic effects [15,16,17,18]. We recently found that in contrast to that of small HBS, the expression of LHBS is maintained in HCC and likely plays an important role in promoting tumor progression [15]. It was found that the level of the major surface protein was lower in tumor samples than in the peritumor samples, in which the HBS signals were usually intense, suggesting that HBS gene expression was downregulated during carcinogenesis. However, in the tumor regions, similar to the nontumor regions, LHBS exhibited strong staining signals in most HCC cases, indicating that LHBS is continuously expressed in carcinogenesis and likely plays an important role in cancer progression [12,16,17]. It was reported that LHBS transgenic mice developed HCC, which suggested that LHBS is uniquely associated with HBV-related carcinogenic processes and the progression of liver cancer [18]. Therefore, it is likely that serum LHBS may also be related to the progression of liver cancer. LHBS is translated from the HBS gene, which produces three different surface proteins; thus, the detection of LHBS depends on protein assays rather than genetic testing. Therefore, we performed in-house development of a monoclonal antibody for the pre-S1 region and developed it into an LHBS ELISA kit. We found that the serum LHBS level is consistent with the liver cancer tissue LHBS level and can be considered an independent risk factor for liver cancer recurrence after surgery.

## 2. Materials and Methods

### 2.1. Patients

Fifty-three patients with HBV-related HCC who underwent hepatectomy between 2010 and 2017 and were being followed up at NCKU Hospital were recruited for this study. All patients were negative for HCV infection. The serum of these patients was tested for various HBV biomarkers, including HBsAg, DNA titer, and LHBS, which was tested by the assay developed in this study. Serum samples were obtained at the time of hepatectomy then analyzed for HBV viral load, HBsAg concentration, and pre-S mutant status. Archival paraffin blocks containing HCC and nontumor liver tissue were retrieved from the Department of Pathology at NCKUH and were subjected to histopathological analysis and to immunohistochemical analysis of LHBS and HBS expression. Sixty-five hepatitis B patients—33 HBeAg(+) patients and 32 HBeAg(−) patients—were also recruited for comparison. This study protocol was approved by the Human Experiment and Ethics Committee of NCKUH with the Institutional Review Board (ER-105-142, 08/01/2017). All the patients provided signed informed consent to use their surgical specimens for this research.

### 2.2. Generation and Purification of the Monoclonal Antibodies Specific for LHBS Pre-S and S Regions

**Figure 1:**
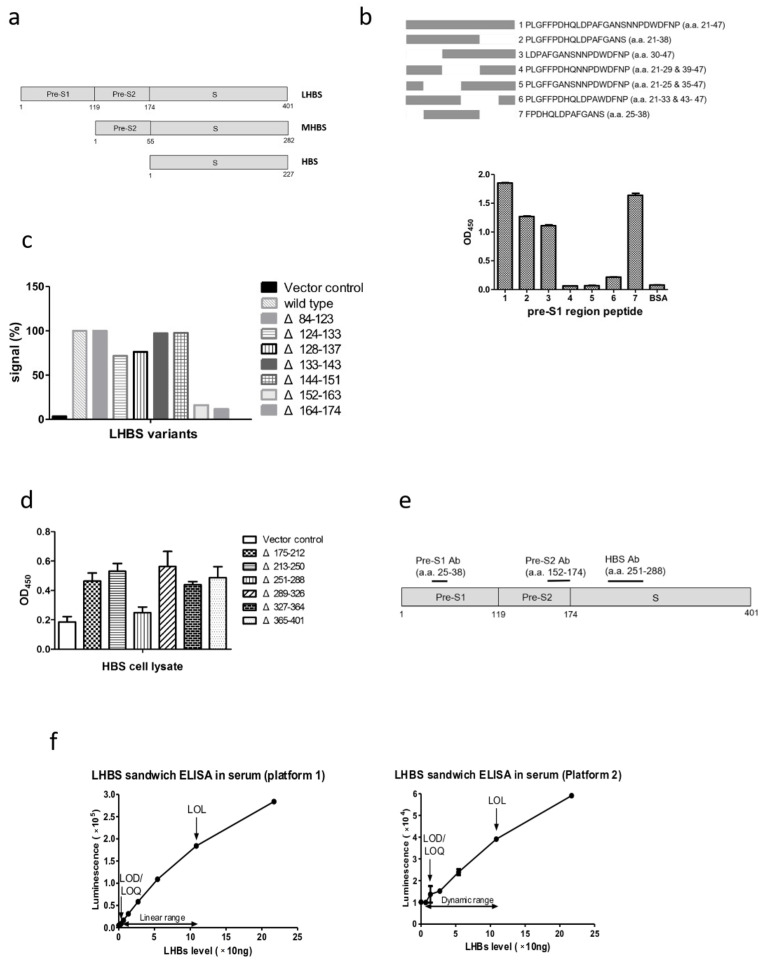
The recognition protein regions of the pre-S1, pre-S2, and HBS monoclonal antibodies. (**a**) Schematic diagrams of the large (L) HBS, medium (M) HBS, and HBS proteins. LHBS includes the pre-S1 (119 a. a.), pre-S2 (55 a. a.), and S (227 a. a.) protein regions. MHBS includes the pre-S2 and S regions, whereas HBS is comprised of the S region only. (**b**) The binding affinities of the pre-S1 antibody to the various oligopeptides spanning the pre-S1 amino acid 21 to 47, previously shown to be highly antigenic. Top: diagram of the full-length oligonucleotide and various deletion variants. Bottom: binding affinities of the pre-S1 antibody to the various pre-S1 region oligonucleotides. (**c**) The binding affinities of the pre-S2 antibody to the LHBS truncated with various pre-S2 regions. Protein lysates of the 293T cells expressing various pre-S2 truncated LHBS were tested for their recognitions by the pre-S2 antibody. (**d**) The binding affinities of the HBS antibody to HBS truncated with various regions in the protein. Protein lysates of the 293T cells expressing various truncated HBS were tested for their recognitions by the HBS antibody. (**e**) Diagram for the LHBS recognition regions of the pre-S1, pre-S2, and HBS antibodies. (**f**) Performance of the chemiluminescence ELISA system developed in this study. The LOD, LOQ, LOL, and detection dynamic ranges of LHBS in HBsAg(+) sera are shown. Platform 1 (**left**), which uses the HBS antibody as detection antibody. Platform 2 (**right**), which uses the pre-S2 antibody as detection antibody.

To generate mouse antibodies specifically recognizing pre-S region of the HBV LHBS, the pre-S region gene sequence was cloned into the pET21b vector, and its expression was induced in the *E. coli* BL21 strain using the inducing chemical isopropyl β-D-1-thiogalactopyranoside (IPTG, 0.2 mM). The purified recombinant pre-S region protein was injected into BALB/c mice to produce pre-S-region-specific antibodies. To produce antibodies that recognize HBS, the HBS recombinant protein, purchased from Leadgene, Inc. (Tainan, Taiwan), was injected into mice to produce HBS-specific antibodies (Figure 1a). The immunized mice were sacrificed, and the splenocytes were used for hybridoma preparation. The hybridoma cells that expressed antibodies recognizing the target pre-S and S proteins were further subjected to serial limiting dilution procedures to generate monoclonal-antibody-producing hybridoma clones. The monoclonal antibodies were tested for their sensitivities/specificities for LHBS, HBS, and the pre-S1 region peptide spanning amino acids 21 to 47, the region previously reported to be highly antigenic [19,20]. The hybridoma clones that exhibited satisfactory sensitivities to and specificities for the target proteins were purified and intraperitoneally injected into mice for ascites fluid production. Finally, antibody-rich ascites fluid was harvested, and the monoclonal antibodies were purified using IgG beads. Some aliquots of these antibodies were also directly conjugated with biotin, as they were evaluated as detection antibodies in the sandwich ELISA system.

### 2.3. Determination of Antibody Sensitivities

To determine the antibody titers, 0.1 ng/μL of each antigen protein or peptide was coated onto each well of the 96-well ELISA plate and was then incubated at 4 °C overnight. The next day, the purified monoclonal antibodies were serially diluted 10-fold and added to the ELISA wells pre-coated with antigens prior to standard ELISA washing steps with phosphate buffered saline with Tween^®^ 20 (PBST, pH 7.4). Antigen–antibody complex signals were detected by incubation with anti-mouse IgG secondary antibodies conjugated to horseradish peroxidase (HRP) followed by the chemiluminescent HRP substrate tetramethylbenzidine (TMB), and the signals were measured using an ELISA chemiluminescence reader. Additionally, to determine the antigen detection limits of the antibodies, the various antigen proteins were serially diluted 10-fold and were then coated onto the ELISA wells; antibodies (1:2000 dilution in PBST) were then added to the well, and standard chemiluminescence detection procedures were then performed. The calibration curves were plotted to determine the limit of detection (LOD), limit of quantification (LOQ), linear range, and limit of linearity (LOL) of the various antibodies for their specific antigen proteins [21].

### 2.4. DNA Sequencing of the Antibody Variable Genes

DNA sequencing of the antibody variable (V) genes in the pre-S1, pre-S2, and HBS-antibody-producing hybridoma cell lines was performed by Leadgene, Inc. (Tainan, Taiwan). Antibody monoclonality was confirmed in each cell line, and the DNA sequences of the framework region (FR) and the complementarity-determining region (CDR) in the antibody V region genes were demonstrated. The corresponding amino acid residues in these regions were determined based on the DNA sequences.

### 2.5. The Sandwich ELISA for LHBS Detection

The in-house-developed LHBS-specific antibody (clone N-term-7-21-5), which targets the pre-S1 region, was coated onto the ELISA wells and incubated at 4 °C overnight. The next day, standard normal serum or HBsAg(+) serum (5 μL serum + 98 μL 2% bovine serum albumin/well) was added to the wells. After sequential washing steps, the detection antibodies, which recognized the pre-S2 or HBS region, were added. The LHBS signals were finally visualized by chemiluminescence detection. The level of LHBS in the serum was quantified by plotting the signal values on a standard curve generated using the pre-S region recombinant protein in various concentrations. Moreover, the LHBS ELISA kit and respective antibodies developed in this study have been filed for US patent application (Appl. No. 63/217,300).

### 2.6. Detection of HBV Markers

The serum HBsAg level was detected using the HBsAg Abbott ARCHITECT^®^ assay. The serum HBV viral load was determined using the COBAS^®^ AmpliPrep/COBAS^®^ TaqMan^®^ HBV Test (Roche Diagnostics).

### 2.7. Immunohistochemical Staining and Interpretation

Immunohistochemistry was performed on 5 µm thick formalin-fixed, paraffin-embedded sections with primary antibodies against LHBS (clone N-term-7-21-5) (1:100) and HBS (T9, Thermo Scientific, Waltham, MA, USA) (1:50). Expression was scored according to the percentages of stained hepatocytes or HCC cells. The liver tissue from a known CHB case was used as positive control. For the negative control of the primary antibodies, the pre-immune mouse immunoglobulin was used. Cytoplasmic LHBS or HBS staining, including mild, moderate, and strong staining was considered positive. The procedures were performed with a Benchmark XT autostainer (Ventana Medical Systems, Oro Valley, AZ, USA). Staining in sections was visualized by an aminoethyl carbazole substrate kit (Zymed Laboratory, San Francisco, CA, USA). Counterstaining was carried out with hematoxylin.

### 2.8. Statistical Analysis

Between-group differences were compared by Mann–Whitney U test. The correlations between serum LHBS, HBS, viral load, and liver stain were assessed using Spearman’s rank correlation coefficient. Disease-free survival (DFS) and overall survival (OS) were calculated using the Kaplan–Meier method, and the log-rank test was used to assess the differences between groups. The thresholds of various clinicopathologic factors to divide them into low and high groups were chosen based on the ROC curve analysis. The threshold was basically the whole number closest to the best-fit threshold number. A Cox proportional hazards regression model was used to measure the independence of different factors. Cox regression was performed via forward stepwise analysis, and only the prognostic variables that were significant in the univariate analysis were included in the model. Finally, *p* values less than 0.05 were considered statistically significant.

## 3. Results

### 3.1. Establishment of the Chemiluminescence ELISA Kit for LHBS Quantification in Serum

The monoclonal antibodies generated in this study were analyzed for antigen recognition regions. For the pre-S1 region antibody, various oligopeptides spanning the region in the pre-S1 N-terminal containing amino acids 21 through 47, previously shown to be highly antigenic, were synthesized and tested for their relative affinities for the pre-S1 antibody [19,20]. The results indicated that the anti-pre-S1 antibody specifically recognized the peptide containing amino acids 25–38 in the sequence FPDHQLDPAFGANS compared with the adjacent protein regions (Figure 1b). For the anti-pre-S2 region antibody, lysates of 293T cells transfected with the LHBS gene with partial deletion of various pre-S regions were tested by ELISA for recognition by the antibody. The results showed that the anti-pre-S2 antibody recognized the full-length and different truncations, except that with deletions of a. a. residues 152–163 and 164–174 in the pre-S2 region, indicating that the epitope recognized by the anti-pre-S2 monoclonal antibody was likely located in the region surrounding a. a. 152–174 in the pre-S2 region (Figure 1c). The anti-HBS antibody recognized all LHBS truncations except that with deletion of a. a. 251–288, which was suggested to be the anti-HBS antibody recognition region (Figure 1d,e).

The mouse monoclonal antibodies specific for the following proteins were generated: pre-S1, pre-S2, and HBS. The amino acid compositions of the CDR1 to 3 in the variable regions of these antibodies were determined based on the DNA sequencing analysis (Table 1). The antibodies were first tested for their sensitivities to the recombinant target proteins. The anti-pre-S1 and anti-pre-S2 antibodies recognized the pre-S region *E. coli* recombinant protein well. For the anti-pre-S1 antibody, the LOQ and LOL values were 0.09 and 12.5 ng/mL, respectively, indicating that the dynamic range of detection was 0.09 to 12.5 ng/mL [21]. The anti-pre-S2 antibody also showed high sensitivity to the pre-S region protein, with the LOQ and LOL values of 0.04 and 1.56 ng/mL, respectively. Regarding the anti-HBS antibody, the LOQ and LOL values for the HBS recombinant protein were 0.06 and 50 ng/mL, respectively (Supplementary Figure S1). As these monoclonal antibodies presented with satisfactory sensitivities to and specificities for their target proteins, they were amplified on a large scale by ascites production, purified by the IgG columns, and conjugated to biotin for sandwich ELISA.

The monoclonal antibodies generated in this study were employed to develop a sandwich ELISA to detect LHBS in serum. The anti-pre-S1 antibody, which specifically recognized LHBS, was used as the coating antibody, whereas either the anti-pre-S2 or anti-HBS antibody was used as the detection antibody in the LHBS ELISA. Pooled HBsAg(+) patient sera with known concentrations of LHBS were used as the standard sample to test its performance. The LHBS sandwich ELISA with the pre-S2 detection antibody revealed an LOQ value of 13.5 ng/mL, and the linearity range was 13.5 to 108.5 ng/mL. In addition, the ELISA with the HBS detection antibody revealed an LOQ value of 1.7 ng/mL, and the linearity range was 1.7 to 108.5 ng/mL (Figure 1f). These data demonstrated that the LHBS sandwich ELISA using either the pre-S2 or HBS detection antibody could detect LHBS in serum sensitively.

### 3.2. The Association of LHBS Expression with HBV Replication and HCC Prognosis

**Figure 2:**
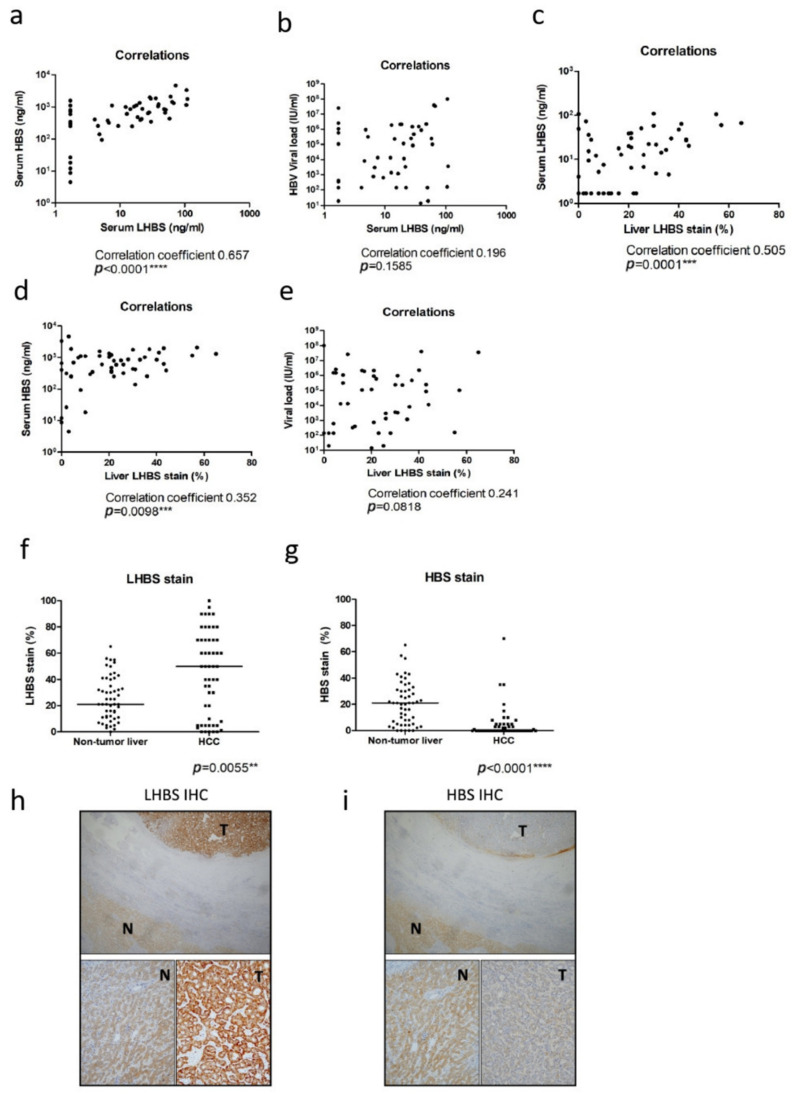
Correlation of the levels of serum and liver LHBS with that of HBS and viral load. (**a**–**e**) Correlation of the serum LHBS with serum HBS (**a**) and viral load (**b**), and liver LHBS stain with serum LHBS (**c**), serum HBS (**d**), and viral load (**e**). (**f**,**g**) Comparison of LHBS stain (**f**) and HBS stain (**g**) between non-tumor liver tissue and HCC tissue. (**h**,**i**) Representative images of LHBS stain (**h**) and HBS stain (**i**) in non-tumor liver tissue (N) and HCC tissue (T). Top panels: gross views of the LHBS and HBS staining. Bottom panels: magnified images of staining results in the tumor (T) and non-tumor (N) regions. LHBS was more diffusely expressed in HCC tissue while HBS expression was diminished in HCC tissue. ** *p* < 0.01, *** *p* < 0.001, **** *p* < 0.0001.

**Figure 3:**
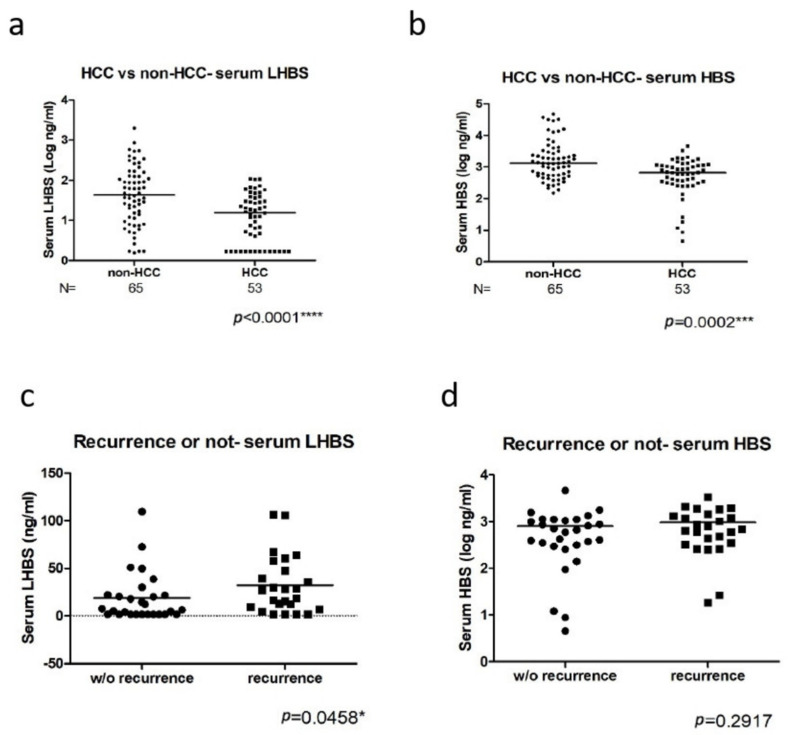
Correlation of the serum LHBS and HBS with HCC incidence and post-surgery recurrence. (**a**,**b**) The LHBS (**a**) and HBS (**b**) levels in CHB and HCC patients. (**c**,**d**) The LHBS (**c**) and HBS (**d**) levels in the HCC patients with or without recurrence. The *p* value for each correlation analysis is shown below the corresponding graph. * *p* < 0.05, *** *p* < 0.001, **** *p* < 0.0001.

Using the LHBS ELISA analysis developed here, the serum LHBS levels in the CHB (*n* = 65) and HCC (*n* = 53) patients were detected. We found that the serum LHBS level was correlated with the HBS but not the viral titer in serum, indicating that HBV surface proteins’ expression does not mainly depend on viral replication status (Figure 2a,b). Moreover, the liver LHBS signals, detected by immunohistochemical staining, showed significant correlations with the serum LHBS and HBS levels (Figure 2c–e). We also found that both serum LHBS and HBS levels were lower in the HCC patients than in the CHB (Figure 3a,b). However, the LHBS expression increased in the tumorous regions, whereas the HBS expression decreased in the tumor, consistent with our previous findings (Figure 2f–i) [15,22,23].

**Figure 4:**
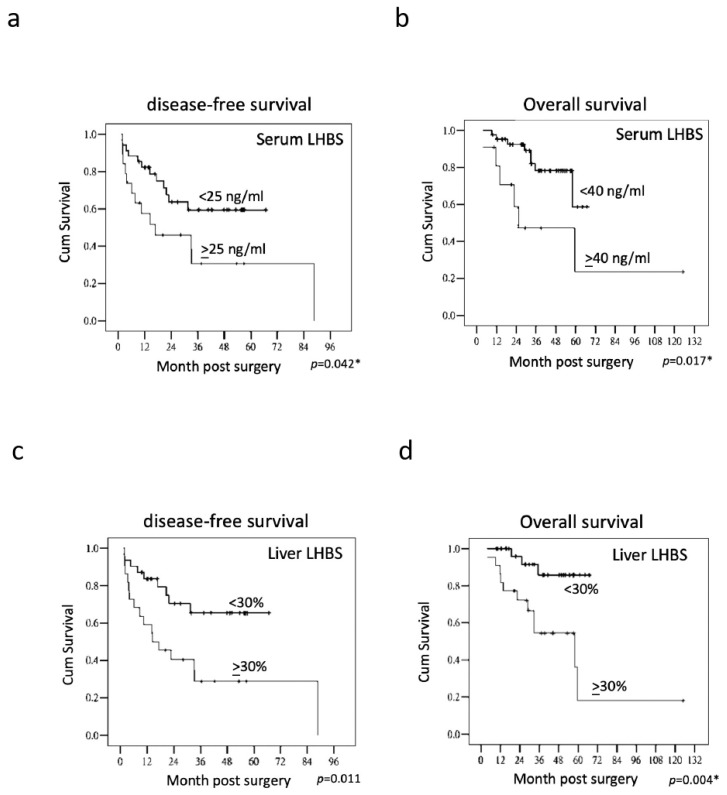
Kaplan–Meier analyses of serum LHBS (**a**,**b**) and liver LHBS stain (**c**,**d**) in disease-free survival and overall survival. The *p* value for each correlation analysis is shown below the corresponding graph. * *p* < 0.05.

We evaluated the association of the serum LHBS with the various clinicopathologic factors in HCC. The serum LHBS but not HBS was correlated with recurrence after surgery (Figure 3c,d). The higher serum LHBS was significantly associated with cirrhosis, advanced AJCC stage, and worse DFS and OS, as determined by the multivariate analysis (Table 2) as well as the Kaplan–Meier analysis with the log-rank test (Figure 4a,b). However, the serum HBS did not significantly correlate with DFS or OS. The immunohistochemistry analysis also revealed that the liver LHBS level was highly correlated with cirrhosis as well as the DFS and OS in the HCC patients receiving surgery. Moreover, the LHBS levels in the tumorous regions were positively associated with vascular invasion (Table 3). In comparison, the liver HBS staining showed a correlation with the AJCC stage but not with any other clinicopathologic parameter analyzed, which suggested that LHBS plays a specific role involving in tumor progression. (Table 4).

Moreover, independent clinicopathologic factors correlated with DFS and OS in HCC patients were estimated by the multivariate analysis. For DFS, the tumor size, vascular invasion, serum LHBS, and liver LHBS staining were significant factors (Table 5). For OS, the multifocal tumor phenotype, tumor size, serum LHBS, and liver LHBS staining were significant (Table 6). On the contrary, neither serum HBS nor liver HBS staining was significantly associated with DFS or OS, indicating that LHBS is an independent parameter associated with HCC recurrence (Table 5 and Table 6).

## 4. Discussion

This study developed a sensitive and simple ELISA method to detect LHBS. With the adaptation of the chemiluminescent substrate in the ELISA, the method allows highly sensitive and quantitative measurement of LHBS [24]. The dynamic range of the measurement is approximately 100-fold, which allows reliable measurement of samples across a wide range of concentrations. With the direct detection of the protein in serum, this detection method is much more time and cost effective than measuring the viral DNA titer. Serum LHBS was better than traditional serum HBS in the stratification of HBe(−) CHB patients and HCC patients, in the correlation of HCC recurrence and in the prediction of DFS and OS in HCC patients after surgery.

LHBS is mainly an integral component of virion envelope and some filamentous SVPs, whereas HBS is expressed in a free form or in spherical SVPs and in the virion in serum [3,25,26,27]. LHBS has been shown to play pivotal roles in the HBV life cycle, including binding to the receptor NTCP, mediating entry into hepatocytes, virus budding, assembly of the envelope and nucleocapsid, and facilitating exocytosis and secretion of the S protein [13,14]. In one study, the composition of HBsAg was shown to have specific patterns across different phases of hepatitis B. Patients in acute or chronic hepatitis phases had significantly lower proportions of LHBS than individuals in the inactive carrier phase irrespective of their HBe antigen status or HBsAg level [28]. Our study also showed that serum LHBS was highest in HBeAg(+) CHB patients, followed by HBeAg(−) CHB patients, whereas HCC patients showed the lowest serum LHBS levels (data not shown). The exact molecular mechanism for the decrease in LHBS in the HCC patients remains unclear. Thus, previous studies reported that the HBV surface proteins generally decrease in the long natural course of HBV infection, possibly due to gradual immune clearance or reduced viral replication after long durations of infection [29,30,31]. In fact, most HBV(+) HCC cases are diagnosed with cancer after many years with CHB, implicating that most HCC cases might have carried the viral infection longer than the CHB cases. Our HCC cohort had a slightly older mean age than the CHB did (HCC 56.3y and CHB 50y), possibly due to that most HCC cases were in the infection for longer than CHB were. In HCC patients, serum LHBS, liver LHBS, and tumor LHBS expression were not significantly correlated with HBeAg status. Serum LHBS and liver LHBS were significantly higher in cirrhotic patients. These findings suggested that LHBS expression is independent of viral replication status when HCC occurs.

LHBS not only plays an important role in HBV infection but also is involved in HCC carcinogenesis [18,32]. In this study, we found that serum and liver LHBS levels were significantly correlated with HCC recurrence. Previous studies have shown that LHBS plays an important role in the development of HBV-related HCC by activating multiple pro-oncogenic pathways. The accumulation of viral LHBS and its prevalent pre-S mutants in chronically HBV-infected hepatocytes triggers a sustained endoplasmic reticulum (ER) overload response, leading to activation of VEGF/Akt/mTOR signaling pathways and IRE1/p38-mediated NF-kB and COX-2 activation [33,34]. LHBS also induces ER stress-mediated Ca^2+^ efflux and ROS generation, which leads to oxidative DNA damage and genomic instability [35]. The pre-S2 mutant protein interacts with c-Jun activation domain binding protein 1 (JAB1), which enhances activator protein-1 transcriptional activity and cell proliferation. Through its binding to JAB1, the pre-S2 mutant protein induces JAB1 nuclear translocation, which activates p27/retinoblastoma/Cdk2/cyclin A pathways and leads to cell cycle progression [36]. Pre-S2 mutant LHBS was also found to block NBS1-mediated homologous recombination repair and induce genomic instability [37]. LHBS elicits hyperploidy by inducing DNA damage and upregulating Plk1, which attenuates cell cycle arrest at the G2/M DNA damage checkpoint [16]. Moreover, recent studies have shown that N-glycans on LHBS are associated with ER stress-mediated cell cycle dysregulation and cell proliferation, suggesting that the post-translational modifications of LHBS play significant roles in regulating HBV-related carcinogenesis [17,38]. An analysis of CHB patients revealed that overexpression of both LHBS and its pre-S mutants is associated with advanced liver disease and HCC development [35,39].

In liver tissue, ground glass hepatocytes identified in patients with HBV-related HCC harbor pre-S deletion variants that largely accumulate in the ER lumen due to mutation-induced protein misfolding and are associated with increased risks of HCC recurrence and metastasis [38,40,41]. Immunohistochemical staining was performed to examine the expression of various surface proteins in tumor tissue surgically resected from patients with HBV-related HCC. HBS protein expression was lower in the tumor than in the non-tumor liver samples, in which the HBS signals were usually intense, suggesting that the HBS gene promoter was downregulated during carcinogenesis. In contrast to that of HBS, the expression of LHBS is maintained in HCC tissue, which likely plays an important role in promoting tumor progression [15]. In this study, we found that serum LHBS was significantly correlated with liver LHBS staining. Serum LHBS is likely a surrogate marker that reflects liver LHBS levels.

In conclusion, we developed a sensitive and simple ELISA method to detect LHBS. Serum LHBS was better than HBS in the stratification of CHB and HCC patients and the correlation of HCC recurrence. Measurement of serum LHBS could be used as a noninvasive test to identify HCC patients with worse DFS and OS, as well as the ones advised to undertake more aggressive treatment modalities or surveillance after surgery.

## Figures and Tables

**Table 1:** Amino acid sequences of the complementarity-determining regions in the variable regions of the monoclonal antibodies developed in this study.

Antibody	CDR Sequences
Pre-S1	*Heavy chain* GYSITSDYA (CDR1)….IRYSGTT (CDR2)….ARGGTGLTY (CDR3)
	*Kappa light chain* ENVGTY (CDR1)….GAS (CDR2)….GQTYNYPFT (CDR3)
Pre-S2	*Heavy chain* GYTFTSYW (CDR1)….INPSNGIT (CDR2)….TIGYDYGSNYEAMDF (CDR3)
	*Kappa light chain* KSLLHSNGITY (CDR1)….QMS (CDR2)….AQNLELPWT (CDR3)
HBS	*Heavy chain* GYTLTDYVIG (CDR1)….EVYPGSVYTSYNEKFKG (CDR2)….AYDGYSPFDY (CDR3)
	*Kappa light chain* KASENVGTYVS (CDR1)….GASNRYI (CDR2)….GQSYNYPHT (CDR3)

**Table 2:** Association of serum LHBS and HBS with various clinicopathologic indicators in the HCC clinical cohort.

Factors	Group	Serum LHBS (ng/mL) Mean (S.D.)	*p* Value	Serum HBS (ng/mL) Mean (S.D.)	*p* Value
Age	<60 years	20.57 (20.33)	0.506	778.94 (559.90)	0.970
	≥60 years	33.04 (38.35)		1030.32 (1199.85)	
Sex	Male	26.00 (27.74)	0.442	929.93 (890.06)	0.275
	Female	20.90 (32.44)		607.32 (568.75)	
Cirrhosis	Absent	14.78 (25.65)	0.005 *	725.67 (807.28)	0.153
	Present	30.77 (28.63)		949.18 (864.96)	
HBeAg	Absent	26.81 (28.46)	0.238	926.86 (935.48)	0.732
	Present	20.10 (28.79)		708.05 (502.38)	
Serum AFP (ng/mL)	<200	25.06 (29.25)	0.687	868.08 (874.88)	0.742
	≥200	24.96 (24.98)		874.53 (691.82)	
Viral load (IU/mL)	<20,000	21.80 (29.65)	0.157	674.75 (871.87)	0.061
	≥20,000	28.96 (26.96)		1103.84 (761.18)	
Tumor differentiation	W	17.84 (9.49)	0.705	634.77 (550.53)	0.471
	M-P	26.32 (30.48)		910.71 (884.07)	
Multifocal tumors	Absent	23.07 (26.56)	0.294	784.99 (664.80)	0.359
	Present	38.01 (38.54)		1421.50 (1564.14)	
Satellite nodule	Absent	25.12 (30.22)	0.566	818.02 (875.01)	0.117
	Present	24.71 (20.14)		1088.51 (689.43)	
Tumor size	<5 cm	23.63 (28.09)	0.684	766.13 (844.84)	0.098
	≥5 cm	27.78 (29.67)		1069.19 (828.50)	
Vascular invasion	Absent	19.34 (23.03)	0.238	684.03 (516.76)	0.242
	Present	31.82 (33.29)		1098.15 (1084.99)	
AJCC stage	I-II	14.79 (16.59)	0.010 *	588.15 (470.62)	0.010 *
	IIIA-C	37.42 (34.63)		1208.49 (1058.58)	
Pre-S deletion	Absent	28.88 (32.94)	0.833	806.16 (832.59)	0.445
	Present	22.52 (25.27)		910.33 (861.86)	

*: *p* < 0.05. Tumor differentiation according to WHO system; AFP, alpha-fetoprotein; AJCC, American Joint Committee on Cancer 2017.

**Table 3:** Association of liver tissue LHBS staining with various clinicopathologic indicators in the HCC clinical cohort.

Factors	Group	Liver LHBS (%) Mean (S.D.)	*p* Value	Tumor LHBS (%) Mean (S.D.)	*p* Value
Age	<60 years	26.85 (16.48)	0.383	41.29 (32.13)	0.264
	≥60 years	23.42 (17.63)		50.68 (31.47)	
Sex	Male	26.85 (16.48)	0.142	41.29 (32.13)	0.205
	Female	23.42 (17.63)		50.68 (31.47)	
Cirrhosis	Absent	18.89 (14.03)	0.044 *	61.31 (27.52)	0.007 *
	Present	29.38 (17.25)		35.35 (30.71)	
HBeAg	Absent	25.89 (17.20)	0.936	43.17 (34.52)	0.578
	Present	24.85 (16.29)		48.78 (23.80)	
Serum AFP (ng/mL)	<200	24.22 (15.68)	0.297	44.37 (32.34)	0.893
	≥200	33.50 (21.70)		46.25 (31.48)	
Viral load (IU/mL)	<20,000	21.93 (14.76)	0.116	42.96 (31.74)	0.641
	≥20,000	30.08 (18.33)		46.70 (32.69)	
Tumor differentiation	W	27.87 (11.24)	0.412	43.12 (24.63)	0.932
	M-P	25.22 (17.69)		44.93 (33.27)	
Multifocal tumors	Absent	24.86 (16.45)	0.478	45.47 (32.32)	0.634
	Present	30.57 (19.67)		39.28 (30.88)	
Satellite nodule	Absent	23.95 (17.10)	0.086	41.67 (31.33)	0.138
	Present	32.80 (14.07)		57.50 (32.85)	
Tumor size	<5 cm	24.71 (15.05)	0.792	46.77 (30.67)	0.509
	≥5 cm	27.38 (20.16)		40.55 (34.76)	
Vascular invasion	Absent	22.37 (16.48)	0.061	34.14 (31.28)	0.009 *
	Present	30.66 (16.28)		57.50 (28.59)	
AJCC stage	I-II	23.93 (16.62)	0.442	43.93 (30.54)	0.809
	IIIA-C	27.66 (17.18)		45.54 (34.15)	
Pre-S deletion	Absent	24.19 (16.14)	0.696	58.09 (29.47)	0.052
	Present	26.56 (17.43)		35.84 (30.76)	

*: *p* < 0.05. Tumor differentiation according to WHO system; AFP, alpha-fetoprotein; AJCC, American Joint Committee on Cancer 2017.

**Table 4:** Association of liver tissue HBS staining with various clinicopathologic indicators in the HCC clinical cohort.

Factors	Group	Liver HBS (%) Mean (S.D.)	*p* Value	Tumor HBS (%) Mean (S.D.)	*p* Value
Age	<60 years	22.70 (16.56)	0.228	3.50 (8.57)	0.177
	≥60 years	17.05 (15.71)		7.10 (16.11)	
Sex	Male	22.90 (16.67)	0.056	3.69 (7.82)	0.980
	Female	11.10 (10.93)		9.50 (22.13)	
Cirrhosis	Absent	17.21 (13.68)	0.358	7.26 (17.45)	0.611
	Present	22.61 (17.54)		3.41 (6.95)	
HBeAg	Absent	21.05 (16.76)	0.920	2.61 (6.51)	0.055
	Present	19.64 (15.65)		10.85 (19.50)	
Serum AFP (ng/mL)	<200	18.91 (14.61)	0.179	4.37 (11.90)	0.223
	≥200	30.62 (22.55)		7.12 (11.78)	
Viral load (IU/mL)	<20,000	17.34 (14.87)	0.113	2.13 (4.54)	0.077
	≥20,000	24.70 (17.42)		8.00 (16.45)	
Tumor differentiation	W	26.75 (11.96)	0.142	10.25 (24.30)	0.687
	M-P	19.60 (16.88)		3.82 (8.03)	
Multifocal tumors	Absent	19.95 (16.11)	0.431	3.82 (7.93)	0.787
	Present	25.42 (18.35)		11.14 (26.12)	
Satellite nodule	Absent	18.41 (15.98)	0.045 *	5.72 (12.94)	0.112
	Present	30.40 (14.87)		0.80 (1.75)	
Tumor size	<5 cm	19.34 (15.09)	0.547	5.34 (13.22)	0.488
	≥5 cm	23.27 (18.71)		3.72 (8.67)	
Vascular invasion	Absent	16.74 (14.39)	0.055	5.66 (14.62)	0.707
	Present	25.87 (17.55)		4.20 (8.38)	
AJCC stage	I-II	18.55 (15.73)	0.287	4.51 (9.43)	0.562
	IIIA-C	23.25 (17.01)		5.12 (14.38)	
Pre-S deletion	Absent	22.00 (17.40)	0.696	7.90 (16.56)	0.207
	Present	19.81 (15.83)		2.75 (6.80)	

*: *p* < 0.05. Tumor differentiation according to WHO system; AFP, alpha-fetoprotein; AJCC, American Joint Committee on Cancer 2017.

**Table 5:** Prognostic significance of clinicopathological indicators, HBsAg expression, and HBV serum profiles for disease-free survival in HCC patients.

Factor	Group	Disease-Free Survival				
		Univariate		Multivariate		
		HR (95% CI)	*p* Value	HR (95% CI)		*p* Value
Age	<60/≥60 years	0.517 (0.205–1.305)	0.163			
Sex	Male/female	0.538 (0.160–1.805)	0.315			
Cirrhosis	−/+	1.121 (0.479–2.621)	0.793			
Serum AFP	<200/≥200 ng/mL	1.570 (0.585–4.213)	0.371			
Viral load	<20,000/≥20,000 IU/mL	2.354 (1.028–5.394)	0.043 *			NS
Differentiation	W/M-P	1.709 (0.508–5.748)	0.419			
Multifocal tumor	−/+	2.056 (0.699–6.044)	0.190			
Satellite nodule	−/+	1.945 (0.768–4.930)	0.161			
Tumor size	<5/≥5 cm	4.140 (1.833–9.352)	0.001 *	3.979 (1.750–9.048)		0.001 *
Vascular invasion	−/+	2.317 (1.023–5.248)	0.044 *	2.590 (1.157–5.797)		NS
AJCC stage	I-II/IIIA-C	2.762 (1.142–6.681)	0.024 *			NS
HBeAg	−/+	1.173 (0.486–2.833)	0.723			
Pre-S deletion	−/+	0.852 (0.379–1.916)	0.698			
Serum HBS	<1000/≥1000 ng/mL	1.806 (0.798–4.088)	0.156			
Serum LHBS	<25/≥25 ng/mL	2.246 (1.007–5.010)	0.048 *	2.267 (1.001–5.137)		NS
Small HBS stain	<30/≥30%	1.686 (0.748–3.802)	0.208			
LHBS stain	<30/≥30%	2.811 (1.229–6.433)	0.014 *	2.650 (1.154–6.087)		0.022 *

*: *p* < 0.05. Tumor differentiation according to WHO system; AFP, alpha-fetoprotein; AJCC, American Joint Committee on Cancer 2017; NS, not significant.

**Table 6:** Prognostic significance of clinicopathological indicators, HBsAg expression, and HBV serum profiles for overall survival in HCC patients.

Factor	Group	Overall Survival				
		Univariate		Multivariate		
		HR (95% CI)	*p* Value	HR (95% CI)		*p* Value
Age	<60/≥60 years	0.453 (0.126–1.624)	0.224			
Sex	Male/female	0.035 (0.000–15.883)	0.283			
Cirrhosis	−/+	1.533 (0.480–4.897)	0.471			
Serum AFP	<200/≥200 ng/mL	0.800 (0.179–3.577)	0.770			
Viral load	<20,000/≥20,000 IU/mL	2.188 (0.730–6.557)	0.162			
Differentiation	W/M-P	1.609 (0.357–7.249)	0.535			
Multifocal tumor	−/+	5.266 (1.549–17.899)	0.008 *	27.399 (4.053–185.239)		0.001 *
Satellite nodule	−/+	1.996 (0.621–6.416)	0.246			
Tumor size	<5/≥5 cm	4.195 (1.398–12.591)	0.011 *	19.935 (3.387–117.335)		0.001 *
Vascular invasion	−/+	2.892 (1.114–7.513)	0.029 *			NS
AJCC stage	I-II/IIIA-C	4.750 (1.314–17.173)	0.017 *			NS
HBeAg	−/+	1.225 (0.376–3.992)	0.736			
Pre-S deletion	−/+	0.911 (0.317–2.615)	0.863			
Serum HBS	<1000/≥1000 ng/mL	2.745 (0.921–8.179)	0.070			
Serum LHBS	<40/≥40 ng/mL	3.508 (1.183–10.406)	0.024 *	6.432 (1.276–32.422)		0.024 *
Small HBS stain	<30/≥30%	2.046 (0.713–5.876)	0.183			
LHBS stain	<30/≥30%	5.431 (1.513–19.497)	0.009 *	5.250 (1.295–21.278)		0.020 *

*: *p* < 0.05. Tumor differentiation according to WHO system; AFP, alpha-fetoprotein; AJCC, American Joint Committee on Cancer 2017; NS, not significant.

## Supplementary Materials

The following supporting information can be downloaded at: https://www.mdpi.com/article/10.3390/ijms23105376/s1.

## Abbreviations

CDR	complementarity-determining region
CHB	chronic hepatitis B
DFS	disease-free survival
ER	endoplasmic reticulum
HBV	hepatitis B virus
HCC	hepatocellular carcinoma
JAB1	c-Jun activation domain binding protein 1
LHBS	large HBV surface protein
LOD	limit of detection
LOQ	limit of quantification
LOL	limit of linearity
NTCP	sodium taurocholate co-transporting polypeptide
OS	overall survival
RFA	radiofrequency ablation
TACE	transarterial chemoembolization
