# Supplementary material for: The Serum Hepatitis B Virus Large Surface Protein as High-Risk Recurrence Biomarker for Hepatoma after Curative Surgery
Source: Int J Mol Sci. 2022 May 11;23(10):5376. doi: 10.3390/ijms23105376 (PMC9140564; doi:10.3390/ijms23105376)
Supplement: Supplementary file 1 [file ijms-23-05376-s001.zip › ijms-1702804-supplementary.pdf]

Supplementary Table S1. Patient profiles of HCC patients (N=53)

variable	
age (mean) (y)	56.3
sex (male/female)	43/10
cirrhosis (no/yes)	19/34
HBeAg (+/-)	14/39
AFP (mean) (ng/ml)	12291.4
viral load (mean) (IU/ml)	4.75×10^6
differentiation (well/moderate/poor)	8/34/11
multifocal tumor (no/yes)	46/7
satellite nodule (no/yes)	43/10
tumor size (mean) (cm)	4.3
vascular invasion (no/yes)	27/26
AJCC stage (I/II/IIIA-IV)	29/14/10

Tumor differentiation according to WHO system; AFP, alpha-fetoprotein; AJCC, American Joint Committee on Cancer 2017.

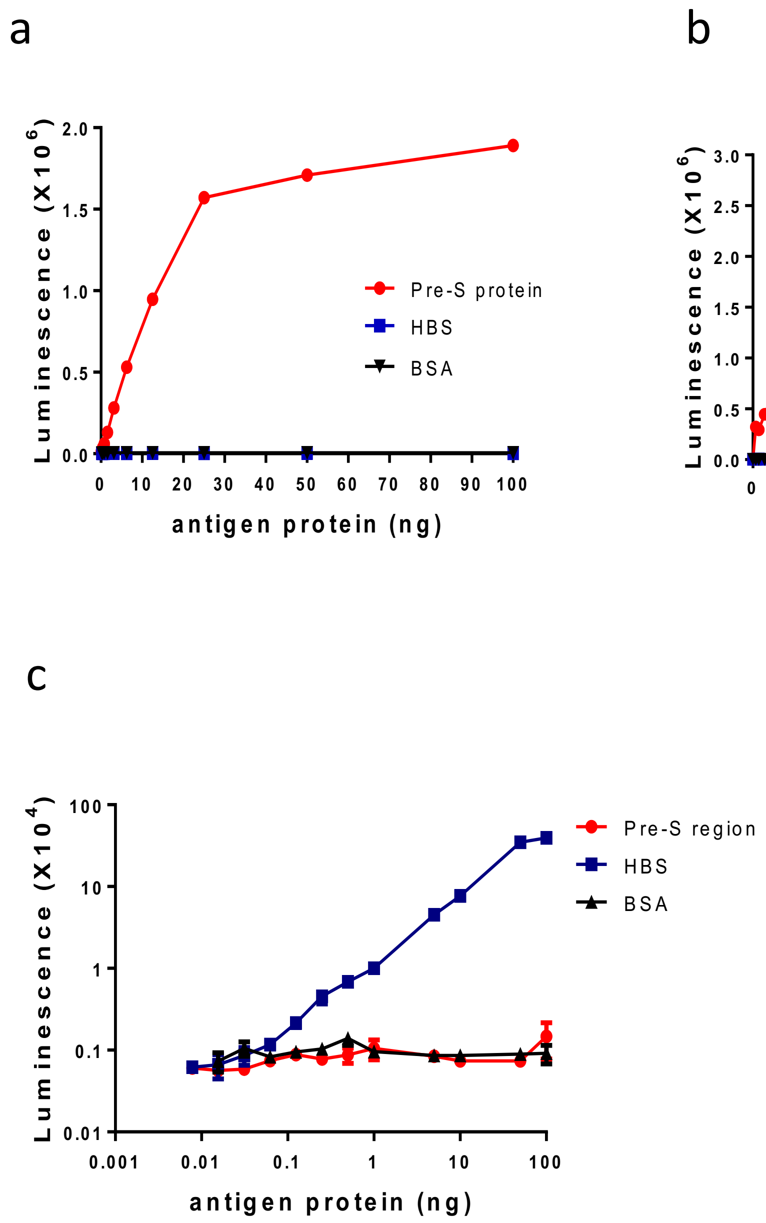

Supplementary Figure S1. Detection sensitivities and dynamic ranges of the monoclonal antibodies for the LHBS chemiluminescent ELISA system. **A-C** The LOD, LOQ, LOL, and detection dynamic ranges of the pre-S1 (**A**), pre-S2 (**B**) and HBS (**C**) antibodies to the respective recombinant antigen proteins were detected.
